# Rubbing time and bonding performance of one-step adhesives to primary enamel and dentin

**DOI:** 10.1590/1678-7757-2016-0627

**Published:** 2017

**Authors:** Maria Paula Jacobucci Botelho, Cristina Pereira Isolan, Júlia Kaster Schwantz, Murilo Baena Lopes, Rafael Ratto de Moraes

**Affiliations:** 1 Universidade Norte do Paraná, Londrina, PR, Brasil; 2 Universidade Federal de Pelotas, Faculdade de Odontologia, Pelotas, RS, Brasil

**Keywords:** Dental bonding, Dental materials, Electron scanning microscopy, Deciduous tooth

## Abstract

**Objectives:**

This study investigated whether increasing the concentration of acidic monomers in one-step adhesives would allow reducing their application time without interfering with the bonding ability to primary enamel and dentin.

**Material and methods:**

Experimental one-step self-etch adhesives were formulated with 5 wt% (AD5), 20 wt% (AD20), or 35 wt% (AD35) acidic monomer. The adhesives were applied using rubbing motion for 5, 10, or 20 s. Bond strengths to primary enamel and dentin were tested under shear stress. A commercial etch-and-rinse adhesive (Single Bond 2; 3M ESPE) served as reference. Scanning electron microscopy was used to observe the morphology of bonded interfaces. Data were analysed at p<0.05.

**Results:**

In enamel, AD35 had higher bond strength when rubbed for at least 10 s, while application for 5 s generated lower bond strength. In dentin, increased acidic monomer improved bonding only for 20 s rubbing time. The etch-and-rinse adhesive yielded higher bond strength to enamel and similar bonding to dentin as compared with the self-etch adhesives. The adhesive layer was thicker and more irregular for the etch-and-rinse material, with no appreciable differences among the self-etch systems.

**Conclusion:**

Overall, increasing the acidic monomer concentration only led to an increase in bond strength to enamel when the rubbing time was at least 10 s. In dentin, despite the increase in bond strength with longer rubbing times, the results favoured the experimental adhesives compared to the conventional adhesive. Reduced rubbing time of self-etch adhesives should be avoided in the clinical setup.

## Introduction

Adhesive materials have been increasingly used to prevent and treat dental caries. However, application time, technical complexity^24^ and unpleasant taste^3^ sometimes are complicating factors for the use of adhesive systems in paediatric dentistry. Self-etch adhesives have been recommended as an alternative to reduce such problems^9^. To be considered ideal, an adhesive system needs, among other features, to be easy to use and to have minimal technical sensitivity^24^. The use of single-step self-etch adhesive systems can save clinical time^9^ and reduce the discrepancy between etched and infiltrated dentin areas, which are commonly associated with etch-and-rinse adhesives^24^. Other advantages of using self-etch adhesives in paediatric dentistry include the fact that the technique does not involve washing and moisture control of the dentin, which are additional steps required in the conventional technique^9^.

Self-etch adhesive systems are composed of hydrophilic monomers, represented mostly by the monomer 2-hydroxyethyl methacrylate (HEMA) and hydrophobic co-monomers, in addition to acidic monomers. The solvent component usually combines water, which is necessary for ionization of the acidic monomers, with ethanol or acetone as co-solvents to increase the vapour pressure of the mixture, which becomes an azeotrope and facilitates evaporation of residual water^17^. The acidic monomer is responsible for etching the dental substrate, creating retention and promoting bonding. It has been shown that the concentrations of acidic monomer and water have significant effects on the aggressiveness and longevity of the bond to enamel or dentin in permanent teeth^16^.

Regarding the method of application of self-etch adhesive systems, it is known that their active application (i.e. with rubbing motion) increases the bond strength and interactions with enamel^5^ and dentin^2^ ^,^ ^12^. The bonding process involves the removal of calcium phosphate from both the enamel and the dentin, which creates surface micropores. These micropores allow the formation of an interdiffusion zone between the enamel and the hybrid layer of the dentin^21^ ^,^ ^24^. The homogeneous impregnation and interpenetration of monomers on the surface of the demineralised tissues are extremely important for the success of the bonds^26^. Primary dentin has lower concentrations of calcium and phosphate than permanent dentin^15^. Thus, etching times for primary dental tissues are usually shorter than permanent teeth^19^, although bond strengths tend to be lower in primary than in permanent dentin^23^. Regarding enamel, despite its lower mineral content in primary teeth^27^, there seems to be no significant difference in etching patterns between primary and permanent teeth^13^.

Recently, single-step adhesives have gained increased attention in dentistry. However, studies usually concentrate on the bonding performance to permanent dental tissues and the effect of adhesive formulation variables and application techniques on the bonding ability to primary dental tissues is seldom reported. Thus, the aim of this study was to investigate whether increasing the concentration of acidic monomers in one-step adhesives would allow a reduction in the rubbing time without interfering with the bonding ability. The hypothesis tested was that shorter application times would have the same bonding potential when the acidic monomer concentration in the adhesive was increased.

## Material and methods

### Experimental design and sample size calculation

This *in vitro* study involved a 3x3x2 factorial design. The factors under study were the concentration of acidic monomer in experimental one-step self-etch adhesives (three levels: 5, 20, or 35 wt%), dental substrate (two levels: primary enamel or dentin), and application time (active rubbing) of the adhesive (three levels: 5, 10, or 20 s). An additional reference group for each dental substrate was treated with a commercial adhesive. Dental hemisections were obtained from primary molars, generating a total of 100 enamel and 100 dentin specimens (n=10 for each group). The sample size was calculated considering the comparative design of nine groups with a 3.8 MPa mean difference in bond strength between groups and 2.2 standard deviation^22^, with a=0.05 and a test power of 0.8. The response variables were bond strength to enamel and dentin (MPa) and failure modes. Scanning electron microscopy (SEM) was used to observe the morphology of the treated dental surfaces.

### Collection and storage of primary teeth

Primary molars were obtained after approval of the research protocol by the local Research Ethics Committee (protocol no 212012). The primary molars used in this study were free of caries or structural defects and were naturally exfoliated or extracted due to orthodontic reasons. The specimens were disinfected by storage in a 0.5% chloramine-T solution for seven days; afterwards, the teeth were cleaned with curettes for removal of the periodontal ligament and were brushed with a dental brush. The teeth were then stored in distilled water at 4°C until use. At least 50 primary teeth were needed. Each tooth was divided into two hemisections, separating the mesial and distal portions of the tooth, to increase the number of test surfaces and decrease the number of teeth needed.

### Formulation of experimental one-step self-etch adhesives

Three one-step self-etch adhesives were prepared by mixing a hydrophobic methacrylate monomer (bisphenol-A glycidyl dimethacrylate - Bis-GMA), a hydrophilic monomer (HEMA), an acidic monomer (1,3-glycerol dimethacrylate phosphate - GDMA-P), solvents (water and ethanol), a photosensitizer (0.4 wt% camphorquinone) and a co-initiator (0.8 wt% 4-dimethylaminoethyl benzoate). This composition reflects a typical formulation of one-step dental adhesives. All monomers were obtained from Esstech Inc. (Essington, PA, USA), except for GDMA-P that was synthesized as described in a previous study^16^. The concentration of HEMA and GDMA-P varied according to the adhesive tested, as shown in Table 1. The adhesives were prepared using two distinct bottles (A and B), which were mixed before application. The concentration of acidic monomer in the mixed adhesives was 5 wt%, 20 wt%, and 35 wt%, thus the materials were labelled as AD5, AD20, and AD35. The pH of the mixed adhesives (n=3) was measured using a digital pH meter (model An2000 - Analion; Ribeirao Preto, SP, Brazil). The formulations were based on a previous investigation^6^ and pilot studies.

**Table 1:** Compositions of the experimental single-step adhesives tested (wt%)

Reagent	AD5			AD20			AD35		
	Bottle A	Bottle B	A+B	Bottle A	Bottle B	A+B	Bottle A	Bottle B	A+B
GDMA-P	10%	-	5%	40%	-	20%	70%	-	35%
HEMA	65%	15%	40%	35%	15%	25%	5%	15%	10%
Bis-GMA	10%	50%	30%	10%	50%	30%	10%	50%	30%
Water	-	20%	10%	-	20%	10%	-	20%	10%
Ethanol	15%	15%	15%	15%	15%	15%	15%	15%	15%
pH			1.91			1.25			1.05

GDMA-P: 1,3-glycerol dimethacrylate phosphate; HEMA: 2-hydroxyethyl methacrylate; Bis-GMA: bisphenol-A glycidyl dimethacrylate. In each bottle A and B, 0.4% of camphorquinone (photoinitiator) and 0.8% of 4-dimethylaminoethyl benzoate (co-initiator) were added in relation to the monomer content

### Application of the adhesives

The hemisections of the primary teeth were embedded in epoxy resin with the buccal or lingual surfaces uncovered. The uncovered surface was lightly wet-polished with 600-grit SiC abrasive papers (Norton; Guarulhos, SP, Brazil) to create a plain surface and remove the aprismatic enamel layer (if any). For each adhesive, we used 30 hemisections, divided randomly into three different rubbing times (n=10 hemisections *per* group): 5, 10 and 20 s. The adhesives were actively applied (with rubbing motion) to enamel surfaces using microbrushes for the corresponding time for each group. The solvent was evaporated for 10 s with compressed air. After testing the enamel surfaces and classifying the failure modes, the same teeth were further wet-polished with 600-grit SiC abrasive papers until medium dentin was exposed. The dentin specimens were randomly divided again into groups, and the adhesives were applied the same way described for enamel. Two additional groups were obtained, testing a conventional etch-and-rinse, two-step adhesive (Adper Single Bond 2 - SB2 - 3M ESPE; St. Paul, MN, USA). In these groups, the enamel was etched for 30 s and the dentin for 15 s using 37% phosphoric acid, followed by application of the adhesive according to the manufacturer's instructions. SB2 is a Bis-GMA/UDMA/HEMA-based adhesive with ethanol and water as solvents and 4.1 measured pH.

### Bond strength test and failure mode analysis

Immediately after application of the bonding systems and solvent evaporation, elastomer moulds with two cylindrical orifices (diameter 1.5 mm, thickness 0.5 mm) were positioned over the surfaces. After placing the moulds, the adhesive was light-cured for 10 s with a light-emitting diode curing unit with 1100 mW/cm^2^ irradiance (Radii-Cal - SDI; Bayswater, Victoria, Australia), allowing delimitation of the bonded area. The orifices were filled with composite resin (Filtek Z250 - 3M ESPE), which was photoactivated for 20 s. The specimens were stored in distilled water at 37°C for 24 h and then were randomly tested under shear stress in a calibrated mechanical testing machine (model DL500 - EMIC; Sao Jose dos Pinhais, PR, Brazil). A stainless steel wire (0.2 mm in diameter) was looped around each cylinder and aligned with the bonded interfaces. The shear bond strength test was carried out with at a crosshead speed of 0.5 mm/min until failure. The operator of the testing machine was blinded to the tested groups. Fractured specimens were observed under x40 magnification using a stereomicroscope (model M125C- Leica Microsystems Inc.; Buffalo Grove, IL, USA) to determine the failure mode: adhesive (interfacial) or mixed failure (partially adhesive and partially cohesive within enamel or dentin). For each hemisection, one resin composite cylinder was obtained and tested (n=10 *per* group). In case of premature failure, the hemisection was eliminated and replaced by a new specimen.

### Statistical analysis

Bond strength data were subjected to a two-way analysis of variance (ANOVA) (acidic monomer concentration x rubbing time). One-way ANOVA was performed to compare the bond strength to enamel or dentin of the experimental and commercial adhesives. For this additional analysis, data from the experimental adhesives included were restricted to the acidic monomer concentration vs. rubbing time groups with highest bond strengths. All pairwise multiple comparison procedures were carried out using the Student-Newman-Keuls’ test (α=0.05). The analyses were performed using the SigmaStat 3.5 software (Systat Software Inc.; Chicago, IL, USA).

### SEM analysis

Additional primary enamel and dentin specimens for each group (n=3) were treated with the adhesives and coated with resin composite as described before. The bonded specimens were embedded cross-sectionally in epoxy resin. Wet-polishing with 1200-, 1500-, 2000-, and 2500-grit SiC abrasive papers was performed, followed by polishing using diamond suspensions (MetaDi - Buehler; Lake Bluff, IL, USA) with 3, 1, and 0.25 um particles. The surfaces were etched with 50% phosphoric acid solution for 5 s and deproteinised by immersion in 2.5% NaOCl solution for 10 min. Specimens were ultrasonically cleaned with distilled water and stored in a container with silica gel for 2 h, at room temperature. The cross-section profiles were coated with gold-palladium alloy and examined by SEM at 15 kV (model JSM-6610 - JEOL Ltd.; Tokyo, Japan).

## Results

**Figure 1:**
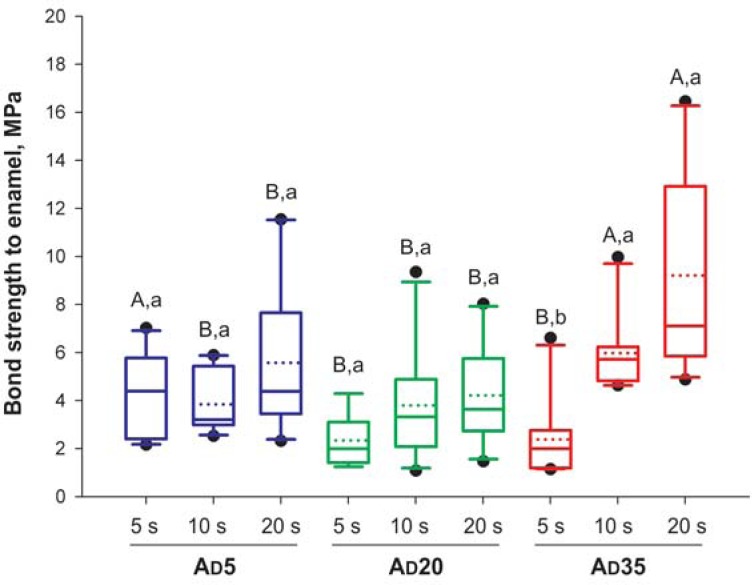
Results for enamel bond strength (n=10). Solid and dotted horizontal lines within each box indicate the medians and means, respectively. Uppercase letters indicate differences among materials; lowercase letters indicate significant differences among rubbing times (p<0.05)

**Figure 2:**
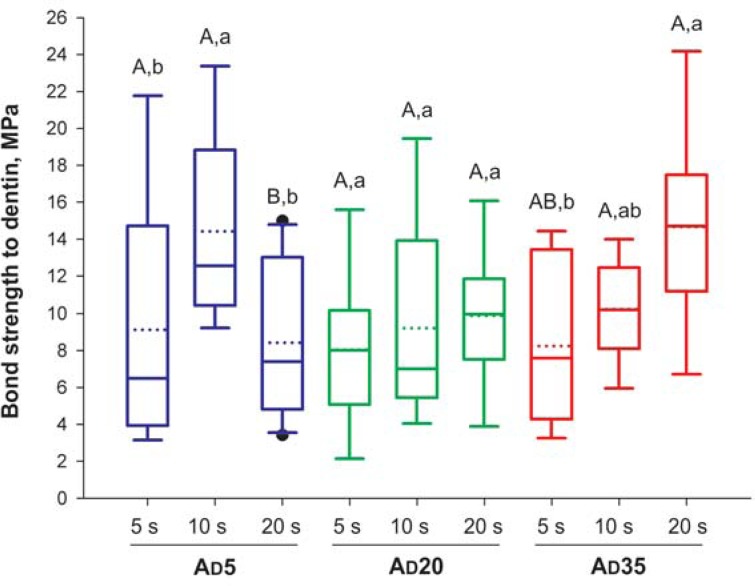
Results for dentin bond strength (n=10). Solid and dotted horizontal lines within each box indicate the medians and means, respectively. Uppercase letters indicate differences among materials; lowercase letters indicate significant differences among rubbing times (p<0.05)

For enamel, the factors “acidic monomer concentration” (*p* =0.004) and “rubbing time” (*p*<0.001) were both significant, as well was the interaction between factors (*p*<0.001). The power of the performed test was ≥0.804. In contrast, for dentin, only the interaction between factors was significant (*p*=0.032), while the factors alone were not (*p*≥0.08). However, the power of the performed test was <0.8 for the factor “acidic monomer concentration”. As shown in Figure 1, increasing the acidic monomer concentration to 35% resulted in significantly higher bond strength when the adhesive rubbing time was at least 10 s. For the other concentrations, rubbing time had little influence on enamel bond strengths. There were no differences between the rubbing times for groups AD5 and AD20, while for group AD35, a rubbing time of 5 s led to a significantly lower bond strength than any other rubbing time. For dentin (Figure 2), increasing the acidic monomer concentration to 20% and 35% led to higher bond strength only when the materials were applied for 20 s. Comparing the different rubbing times in dentin, application for 20 s resulted in significantly lower bond strength than the other times for group AD5, but significantly higher bond strength for group AD35.

**Figure 3:**
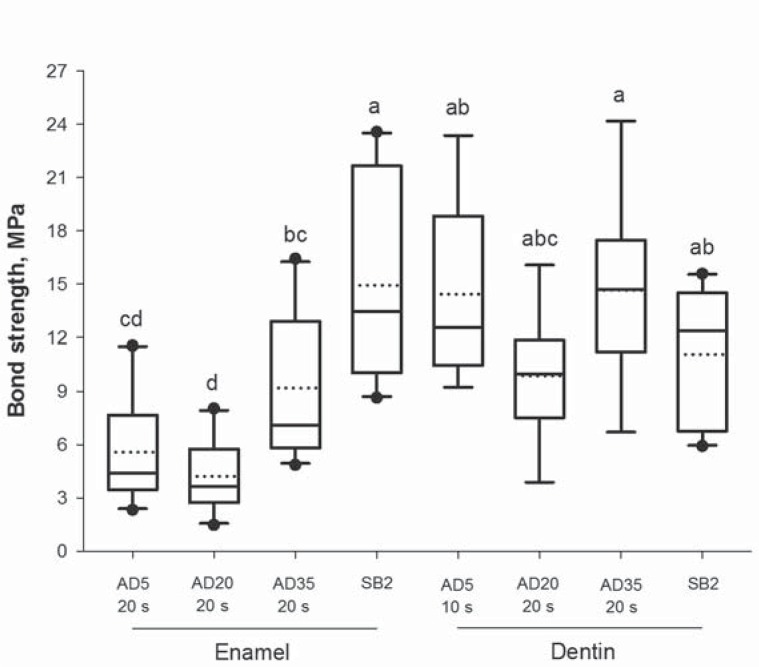
Results for enamel and dentin bond strengths of the commercial adhesive and the adhesive-rubbing time combinations with the highest means for each substrate (n=10). Solid and dotted horizontal lines indicate medians and means, respectively. Distinct letters indicate significant differences among groups (p<0.05)

Figure 3 shows the comparisons of bond strength to enamel and dentin for the experimental groups and the commercial material. For enamel, the commercial adhesive showed significantly higher bond strength than the experimental materials, while for the dentin, no significant difference was observed. In this study, in general, dentin bond strengths of the experimental adhesives were significantly higher than enamel bond strengths. Results for the failure analysis are presented in Table 2. Adhesive failures predominated in both substrates. While only a few failures in enamel were classified as mixed, especially for the commercial group, failures in dentin were mostly mixed. Failure modes were not influenced by either the acidic monomer concentration or the rubbing time.

**Table 2:** Frequencies of failure modes for all groups

Substrate	Material	Rubbing time	Failure modes %	
			Adhesive	Mixed
Enamel	AD5	5 s	100	-
		10 s	100	-
		20 s	100	-
	AD20	5 s	100	-
		10 s	100	-
		20 s	100	-
	AD35	5 s	100	-
		10 s	100	-
		20 s	100	-
	SB2		60	40
Dentin	AD5	5 s	100	-
		10 s	90	10
		20 s	100	-
	AD20	5 s	70	30
		10 s	80	20
		20 s	80	20
	AD35	5 s	90	10
		10 s	100	-
		20 s	80	20
	SB2		90	10

Adhesive: failure at the enamel or dentin interface; mixed: remnants of composite resin left on the dental surface

**Figure 4:**
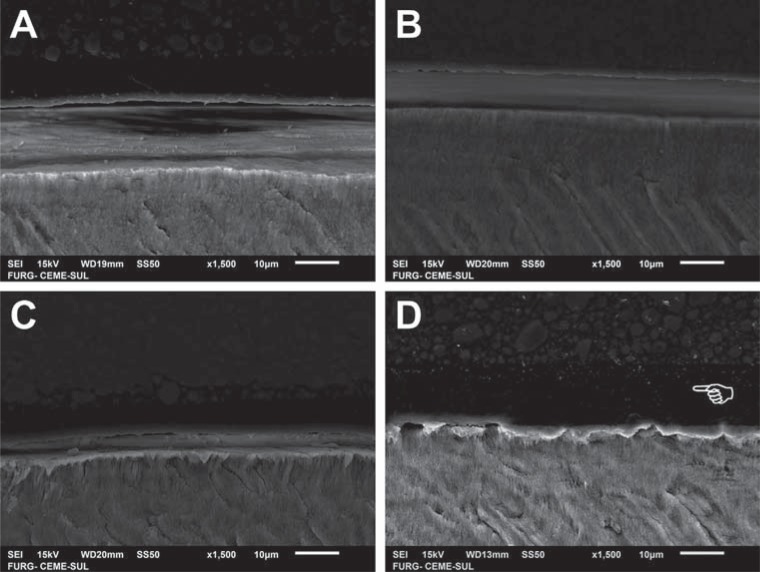
Scanning electron microscopy (SEM) images of enamel bonded interfaces of groups presented in Figure 3 - A: AD5 applied for 20 s; B: AD20 applied for 20 s; C: AD35 applied for 20 s; D: commercial etch-and-rinse adhesive. The pointer indicates the thicker adhesive layer observed for the commercial material

**Figure 5:**
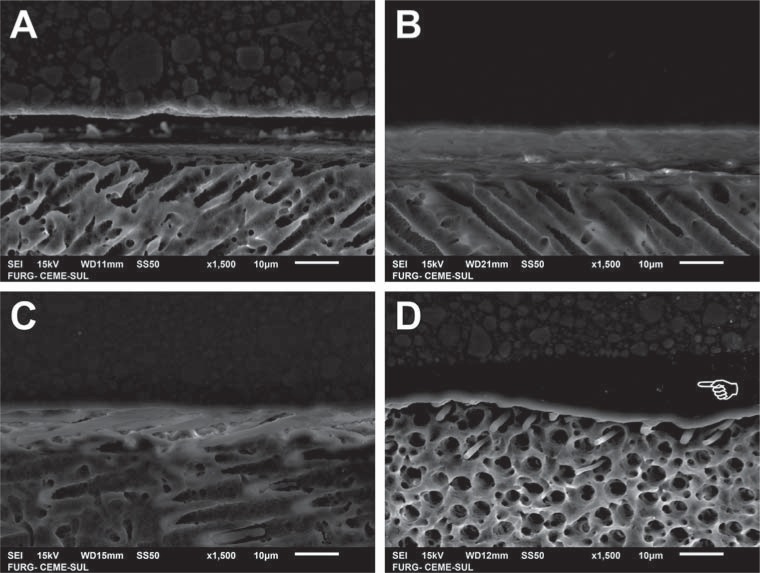
Scanning electron microscopy (SEM) images of dentin bonded interfaces of groups presented in Figure 3 - A: AD5 applied for 10 s; B: AD20 applied for 20 s; C: AD35 applied for 20 s; D: commercial etch-and-rinse adhesive. The pointer indicates the thicker and more irregular adhesive layer observed for the commercial material

SEM images of the bonded interfaces of groups presented in Figure 3 are shown in Figure 4 (enamel) and Figure 5 (dentin). The differences are not appreciable among the experimental materials with distinct acidic monomer concentrations. The adhesive layer was thicker (Figure 4D and Figure 5D) and more irregular (Figure 5D) for the commercial etch-and-rinse compared with the self-etch adhesives. More resin tags in dentin seemed to be formed for AD35 (Figure 5C) compared with the other experimental adhesives, but no other clear differences in interfacial morphology were noticed.

## Discussion

Results of this study indicate that both acidic monomer concentration and rubbing time can influence the bonding potential of one-step self-etch adhesives to primary dental tissues. The substrates tested (enamel or dentin) also had a major role on the bonding performance. Overall, an increase in acidic monomer concentration led to improved bond strength only when the rubbing time of the adhesive was at least 10 s. Therefore, the hypothesis tested cannot be accepted.

The increase in acidic monomer concentration led to a reduction in pH of the adhesives. Although the three experimental adhesives were classified as intermediately strong^24^, the adhesive AD35 had a pH value close to strong aggressiveness. This information is important because previous studies have indicated that self-etch adhesives with intermediate aggressiveness and particularly mild aggressiveness tend to present longer-lasting bonds^8^, i.e., water degradation effects are lower when compared with more acidic materials. The explanation for this finding is that the acidity of the adhesive affects the hydrophilicity of the material and consequently the permeability and susceptibility to hydrolysis of the adhesive layer. Moreover, a less severe demineralisation allows more collagen to remain protected by hydroxyapatite, which is less susceptible to water degradation^18^. Interestingly, the differences in pH did not generate appreciable differences in the morphology of the bonded interfaces. In the first generation of self-etch adhesives, the content of acidic monomers tended to be higher as compared with current formulations because only with strong self-etch adhesives the typical resin tags observed in bonded dentin are formed^24^. Currently, it is known that resin tags do not contribute significantly to dentin bonds and that the type of acidic functional monomer is of greater importance than its concentration.

The bond strength results indicate that the adhesive potential of the experimental adhesives varied with the material tested, the rubbing time, and the substrate type. Thus, explanations for the findings should rely on several combined mechanisms. The acidic monomer concentration tends to increase the adhesive aggressiveness, enhancing the dissolution potential of the hydroxyapatite present in enamel and dentin, and the differences in mineral content in the substrates affect the bonding mechanism differently. Greater surface dissolution, though positive, needs to be accompanied by effective infiltration of the adhesive components into the dental tissues and effective polymerisation *in loco*. The presence of a larger amount of acidic monomers can interfere with the adhesive polymerisation because methacrylate monomers with a terminal acid radical can react with the free radicals generated during radicular polymerisation and reduce the degree of C=C conversion^1^. Moreover, reduced pH increases the difficulty in removing ethanol and water during solvent volatilisation^28^, which can also affect polimerisation^20^. The combination of all these aspects have an impact on the bonding performance. In any case, proper volatilization of the solvent and photoactivation of the adhesive layer are essential steps for the application of simplified bonding systems and should not be neglected during clinical application.

Alteration in the water concentration of self-etch adhesives may be enough to increase the etching aggressiveness of primary enamel^13^. In primary teeth, the minimum concentration of water needed to cause sufficient enamel demineralisation found in a previous study was 20%^13^. Higher water concentration may hinder its elimination by evaporation^7^. Another study suggested that the water fraction needs to be sufficient for adequate ionization of acidic monomers but the concentration of the monomers cannot be altered to avoid negative influence on dentin bonds^14^. In this study, the concentrations of water and solvent were standardized, and the concentration of GDMA-P was altered by reducing the HEMA content.

The commercial adhesive showed higher bond strength to primary enamel than experimental self-etch adhesives. This result corresponds with those of a previous study^10^ indicating that conventional adhesives have higher capacity to bond to enamel than self-etch adhesives due to the higher demineralisation capacity of phosphoric acid compared to acidic monomers. Acid etching before application of dental adhesives still is considered the gold standard technique for bonding to enamel. Nonetheless, in dentin, the bond strength of the commercial adhesive was equivalent to the self-etch adhesives, despite the absence of acid etching for the experimental groups. This result reinforces the fact that phosphoric acid applied to dentin does not benefit adhesion potential^24^ because the dentin does not need to be completely dissolved for hybridisation to occur. Moreover, this result confirms that one-step self-etch materials may have similar bonds to enamel and dentin compared with etch-and-rinse adhesives.

For the self-etch adhesives tested, the bond strength to dentin was higher to that of enamel. This result is related to the previously mentioned fact that, in dentin, hybridisation does not depend on an extensive dissolution of mineral content or deep micromechanical imbrication. In contrast, a recent study observed similar dentin bond strengths between etch-and-rinse and self-etch adhesives^25^. The bonding of self-etch adhesives to dentin occurs through superficial hybridisation, without removal of the smear layer^6^, and chemical bonding of the acidic monomers to the hydroxyapatite^11^. Similar bonded morphology observed for different self-etch adhesives highlights the role of chemical interaction in generating different bonding abilities. The increase in concentration of acidic monomers could have a positive effect on the longevity of the bond to enamel and dentin due to a higher chemical affinity, an effect that needs to be further investigated. As the bonding to enamel is mainly due to mechanical interlocking caused by the diffusion and polymerisation of resin monomers on the etched surface^13^, the failure patterns observed with the tested adhesives suggest that such interlocking was incomplete.

For this study, primary molars were chosen because they present a enamel mineralisation that is more uniform throughout the entire surface, which does not occur with primary canines or incisors^27^. Only healthy teeth were used because carious teeth are not considered ideal models for comparisons of the micromorphology of the dentin-resin interface^19^. The tested hypothesis was rejected, as it was not possible to reduce the application times of self-etch adhesives by only increasing the concentration of acidic monomers. However, it is interesting to note the comparison of the experimental groups with the commercial reference in dentin. The bonding to dentin depends on the hybridisation or infiltration of resin to the collagen fibrils network^26^. The use of self-etch adhesives decreases the possibility of creating etched but non-infiltrated areas because the monomer infiltration occurs simultaneously with the surface conditioning^21^. In primary teeth, more dentin demineralisation is expected through the action of conditioning acids, which suggests that a shorter rubbing time is desirable^19^. Although the minimum time needed for conditioning the primary dentin is 15 s with the use of phosphoric acid^4^, 5 s of rubbing time of the experimental adhesive system with 20% GDMA-P led to bond strength values similar to those obtained in the commercial group. Therefore, these results encourage future investigations on the performance of simplified adhesive materials applied to primary substrates.

Due to the high costs and the difficulty in identifying the true causes of failure in adhesive restorations in the clinical environment, *in vitro* studies are common; at times, they are able to forecast the clinical efficacy of materials^24^. However, testing materials in laboratory have limitations in terms of clinical factors that can impact the performance of dental materials. In paediatric dentistry, the presence of moisture during the adhesive procedure due to difficulty in maintaining an effective isolation of the operative field is an example^3^. However, the results of this study suggest that it would be possible to eliminate the clinical step of acid conditioning and washing, at least in primary dentin, which would eliminate the unpleasant taste caused by the acid wash^3^. Further studies, especially related to the morphology of the bonding to primary tissues, could clarify the mechanism of adhesion to these substrates.

Dental adhesive materials are increasingly common in dentistry, whether to prevent caries or to restore carious lesions and fractures. The basic adhesion mechanism to dentin or enamel for either primary or permanent teeth is based on an exchange of substances in which the minerals in the hard tissues are replaced by resinous monomers present in adhesives that bond micromechanically to the porosities created by the acid material. The proportions of minerals are different between primary and permanent teeth, as are the depths of the dentin and enamel. These two substrates have important differences that cannot be overlooked. The use of adhesive materials should consider all of these factors. In paediatric dentistry, the child's age must also be acknowledged. Therefore, the development of an adhesive material that addresses all of these factors needs to be undertaken because, in childhood, dental caries are difficult to control and hard tissue trauma is highly prevalent. In both of these cases, adhesive restorations are common.

## Conclusions

This *in vitro* study indicates that both the acidic monomer concentration present in one-step self-etch adhesives and the rubbing time can influence the bonding performance of the adhesive to enamel and dentin in primary teeth. Overall, increasing the acidic monomer concentration only led to an increase in bond strength to enamel when the rubbing time was at least 10 s. In dentin, despite the increase in acidic monomer concentration that led to an increase in bond strength with longer rubbing times, results favoured the experimental adhesives compared to the conventional adhesive. Thus, reduced rubbing times of self-etch adhesives should be avoided in the clinical setup.
